# Metformin inhibits digestive proteases and impairs protein digestion in mice

**DOI:** 10.1016/j.jbc.2023.105363

**Published:** 2023-10-19

**Authors:** Caleb J. Kelly, Andrew A. Verdegaal, Brent W. Anderson, William L. Shaw, Natasha A. Bencivenga-Barry, Ewa Folta-Stogniew, Andrew L. Goodman

**Affiliations:** 1 Microbial Sciences Institute, Yale University, West Haven, Connecticut, USA; 2 Section of Digestive Diseases, Department of Internal Medicine, Yale University School of Medicine, New Haven, Connecticut, USA; 3 Department of Microbial Pathogenesis, Yale University School of Medicine, New Haven, Connecticut, USA; 4 Keck Biotechnology Resource Laboratory, Yale University School of Medicine, New Haven, Connecticut, USA

**Keywords:** metformin, protease, enteropeptidase, trypsin, maldigestion, protein degradation, intestine, diabetes

## Abstract

Metformin is among the most prescribed medications worldwide and the first-line therapy for type 2 diabetes. However, gastrointestinal side effects are common and can be dose limiting. The total daily metformin dose frequently reaches several grams, and poor absorption results in high intestinal drug concentrations. Here, we report that metformin inhibits the activity of enteropeptidase and other digestive enzymes at drug concentrations predicted to occur in the human duodenum. Treatment of mouse gastrointestinal tissue with metformin reduces enteropeptidase activity; further, metformin-treated mice exhibit reduced enteropeptidase activity, reduced trypsin activity, and impaired protein digestion within the intestinal lumen. These results indicate that metformin-induced protein maldigestion could contribute to the gastrointestinal side effects and other impacts of this widely used drug.

Metformin is the first-line agent for the treatment of type 2 diabetes and one of the most prescribed medications worldwide. There is also growing interest in the potential use of metformin for obesity and some cardiovascular, hepatic, renal, and oncologic diseases (1). Because of the high dose required (commonly 2000 mg/day) and incomplete gastrointestinal absorption, metformin may reach high intestinal concentrations. Observed effects of metformin such as weight loss, diarrhea, altered microbiome, and vitamin B_12_ deficiency are not fully understood but may involve intestinal actions of the drug (2). Adverse gastrointestinal side effects attributed to metformin occur in both diabetic and nondiabetic patients and are observed in ∼25% of patients, resulting in discontinuation in ∼5% (2, 3). The primary mechanism by which metformin lowers blood glucose is the reduction of hepatic gluconeogenesis, which results from the inhibition of key enzymes (4). Because of the potential to inhibit enzymes and high drug concentration within the intestine, we sought to determine if metformin could inhibit digestive enzymes. Metformin inhibition of pepsin, a gastric protease, can occur *in vitro*, but its effects on other pancreatic and intestinal proteases are unknown (5). We present experimental data to support the hypothesis that metformin can inhibit important digestive proteases, which could contribute to both the beneficial and detrimental impacts of this drug in the gastrointestinal tract.

## Results

**Figure 1:**
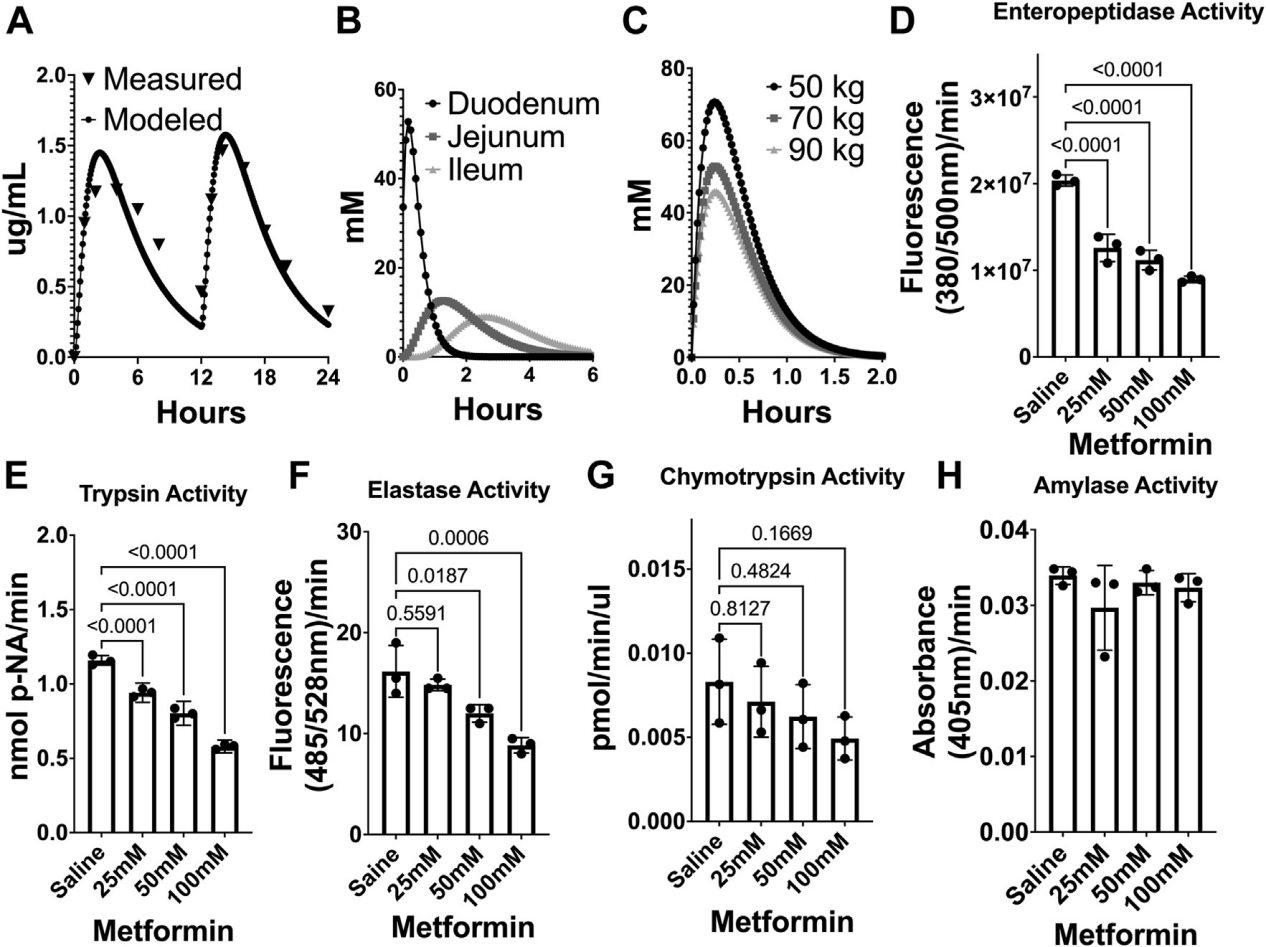
**Metformin selectively inhibits digestive proteases *in vitro***. *A*, pharmacokinetic modeling of plasma metformin kinetics recapitulates measured plasma drug pharmacokinetics in humans. *B*, predicted gastrointestinal intraluminal metformin concentrations after a 1000 mg dose in a 70 kg human. *C*, impact of body mass on predicted duodenal concentration. *D–H*, metformin significantly inhibits enteropeptidase (*D*), trypsin (*E*), and elastase (*F*) activity, but not chymotrypsin (*G*) or amylase (*H*), at concentrations below predicted duodenal levels. *Error bars* indicate the standard deviation from three independent experiments.

To estimate the intraluminal metformin concentration in the human intestine, we first calculated drug properties based on metformin’s 2-dimensional structure. Providing these values in a compartmental model using published values for bioavailability, intestinal permeability, and other parameters (Table 1) reasonably approximated metformin plasma concentrations measured in humans (Fig. 1*A*) (6). This compartmental model predicts that intestinal metformin concentration is highest in the duodenum: in a 70 kg human, the predicted peak concentration exceeds 50 mM after each 1000 mg dose (Fig. 1*B*); in a 50 kg individual, peak duodenal metformin concentration exceeds 70 mM (Fig. 1*C*).

**Table 1:** Pharmacokinetic modeling parameters

Parameter	Value	Reference
Dose (metformin HCl)	1000 mg	(6)
Molecular weight	165.63	ADMET Predictor v10.4
Solubility	100.05 mg/mL	ADMET Predictor v10.4
Mean precipitation time	900 s	ADMET Predictor v10.4
Diffusion coefficient	0.75	ADMET Predictor v10.4
Drug particle density	1.2 g/ml	ADMET Predictor v10.4
Log P	−0.82	ADMET Predictor v10.4
Duodenal permeability	0.27 × 10^4^ cm/s	(19)
Dose volume (water with medication)	250 ml	GastroPlus v9.8.2 Default
Body mass	70 kg	GastroPlus v9.8.2 Default
Duodenal volume	41.56 ml	GastroPlus v9.8.2 for 70 kg mass
Jejunum volume (portion 2 of 3)	122.3 ml	GastroPlus v9.8.2 for 70 kg mass
Ileum volume (portion 3 of 3)	49.83 ml	GastroPlus v9.8.2 for 70 kg mass

We hypothesized that the high concentration of metformin in the duodenum affects the activity of digestive enzymes in this compartment. Enteropeptidase localizes on the apical surface of duodenal enterocytes and has a key role in activating zymogens secreted by the pancreas, including the conversion of trypsinogen to active trypsin. Trypsin then participates in protein hydrolysis directly and activates other zymogens. Notably, 25 mM metformin significantly inhibits the activity of purified enteropeptidase and trypsin, with increasing inhibition in a dose-dependent manner (Fig. 1, *D* and *E*). Metformin inhibits elastase activity at higher concentrations (50–100 mM) (Fig. 1*F*). The drug had no effect on the activity of chymotrypsin or amylase at any of the tested concentrations, suggesting that the inhibitory effect is enzyme selective (Fig. 1, *G* and *H*).

We next focused on enteropeptidase, which is proximal in the protease activation cascade and therefore has the greatest potential to influence protein digestion and was highly sensitive to metformin inhibition. Surface plasmon resonance (SPR) measurements of the interaction between metformin and recombinant human enteropeptidase identified weak but significant direct interactions with a predicted affinity (K_D_) of 129.4 (SD ± 30.9) mM. SPR measurements of direct binding between metformin and amylase (which is not inhibited by the drug) revealed no interaction even at concentrations of 500 mM (Table 2).

**Table 2:** Surface plasmon resonance

Protein	Experimental replicate	Technical replicates	Kd (mM)	Maximum response	Estimated # metformin bound
Amylase	1	6	N/A	N/A	N/A
Enteropeptidase	1	12	151.3	239.5	33.8
	2	12	107.6	149.5	26.6
	Average		129.4	194.5	30.2
	SD (±)		30.9	63.6	5.1

**Figure 2:**
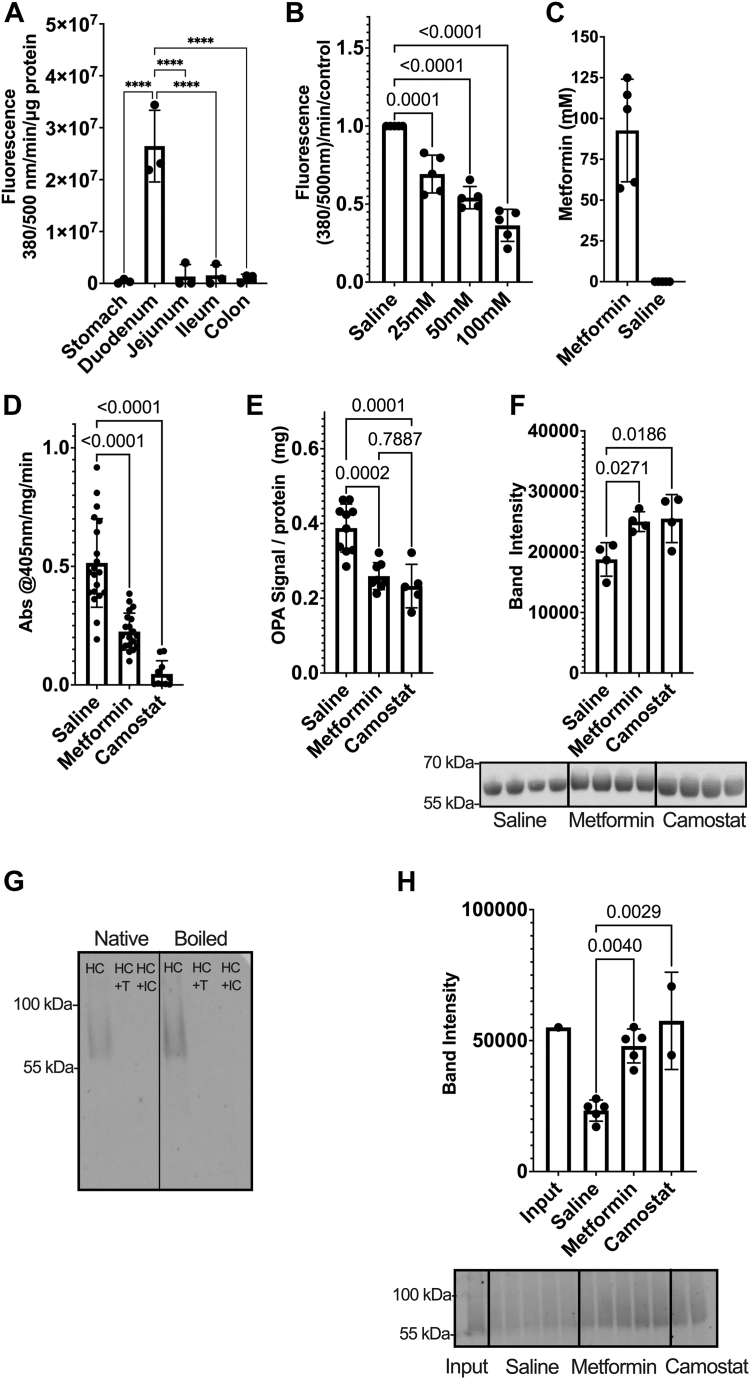
**Metformin inhibits digestive enzyme activity *ex vivo* and *in vivo****. A*, enteropeptidase activity along the length of the murine gastrointestinal tract. *B*, metformin-mediated inhibition of enteropeptidase activity of murine duodenal tissues *ex vivo. C*, duodenal metformin concentration 20 min after administration of 300 mg/kg metformin. *D*, impact of metformin treatment on trypsin activity *in vivo. E*, extent of protein hydrolysis (normalized to total protein) in saline-, metformin-, or camostat-treated mice. *F*, quantification of murine transcobalamin in small intestinal samples. *G*, recombinant human haptocorrin (HC) is a glycoprotein that is degraded by incubation with trypsin (T) or intestinal contents (IC). *H*, quantification of recombinant human haptocorrin after incubation with small intestinal contents from treated animals. Data represent 3 (*A*), 5 (*B* and *C*), 10 to 20 (*D*), 5 to 10 (*E*), and 4 (*F*) mice/group with *error bars* representing the standard deviation. In panel H, data represent separate *ex vivo* incubations with samples from 5 (saline, metformin) or 2 (camostat) mice/group. ∗∗∗∗*p* < 0.0001.

To determine effects of metformin in a murine model, we first mapped the distribution of enteropeptidase activity to the murine duodenum with minimal activity found in other locations (Fig. 2*A*). *Ex vivo* metformin treatment of murine duodenal tissue extracts revealed significant, concentration-dependent inhibition of enteropeptidase activity, suggesting that other duodenal proteins do not ameliorate the inhibitory effect of the drug (Fig. 2*B*). To determine the effect of metformin on protease activity *in vivo*, we treated mice with metformin (300 mg/kg, as described (4, 7)), negative saline control, or the serine protease inhibitor camostat which inhibits enteropeptidase and trypsin (8). Twenty minutes after treatment, metformin averaged 92 mM in the small intestine of metformin-treated animals (Fig. 2*C*). Notably, trypsin activity of the intestinal contents was significantly reduced in samples from mice treated with metformin or camostat compared with saline-treated animals (Fig. 2*D*).

To determine if reduced trypsin activity impacts protein digestion, we next measured the degree of luminal protein hydrolysis using O-phtaldialdehyde (OPA), which produces a signal proportional to the abundance of primary amines (peptide N termini) in a sample (9). Samples from metformin-treated mice exhibited significantly lower OPA signal (normalized to total protein) compared to saline-treated animals, indicating fewer free primary amines and reduced protein hydrolysis (Fig. 2*E*). This reduction in protein digestion was equivalent in animals treated with metformin and animals treated with camostat (Fig. 2*E*).

In addition to its role in dietary protein digestion, gastrointestinal proteases also mediate important vitamin uptake processes in the gut. For example, vitamin B_12_ carrier proteins (transcobalamin in mice, haptocorrin in humans) require tryptic digestion within the intestinal lumen to enable vitamin B_12_ to bind intrinsic factor for absorption in the distal small intestine (10, 11). To investigate whether metformin inhibition of gastrointestinal protease activity can impact the processing of these endogenous protease targets, we first measured the impact of metformin treatment on intestinal transcobalamin levels in mice. Indeed, metformin treatment significantly increased the levels of undigested transcobalamin (normalized to total protein content) compared to the saline control (Fig. 2*F*). The levels of undigested transcobalamin were equivalent in metformin-treated and camostat-treated mice (Fig. 2*F*). Consistent with the impact of metformin treatment on murine transcobalamin levels *in vivo*, incubation of intestinal tissue samples from metformin-treated animals with recombinant human haptocorrin, a glycoprotein degraded by trypsin (Fig. 2*G*), produced limited haptocorrin digestion compared to tissue samples from saline-treated mice (Fig. 2*H*). This suggests that metformin-mediated reduction of protease activity impacts both murine and human vitamin carrier proteins that rely on protein digestion for their function.

## Discussion

Metformin is among the most widely employed medications in terms of number of prescriptions (12); because doses can exceed 2000 mg/day, this drug is likely the single most widely prescribed medication by weight. The large doses required to achieve therapeutic benefit result in micromolar drug concentrations in peripheral tissue and low millimolar concentrations in intestinal tissue (13). In the gastrointestinal lumen, concentrations are likely much higher: indeed, PBPK models that accurately recapitulate serum metformin pharmacokinetics predict that duodenal drug concentration reaches 50 to 75 mM prior to absorption (Fig. 1, *A*–*C*). At these and lower concentrations, metformin inhibits enteropeptidase and trypsin *in vitro* and reduces these enzyme activities in murine duodenal extracts *ex vivo*. In mice, metformin administration significantly reduces trypsin enzyme activity and reduces digestion of both total protein and endogenous vitamin carriers that require protease activity for their function.

SPR measurements indicate that the metformin binds to enteropeptidase directly and with low affinity. Although metformin exhibits no binding to control proteins such as amylase, these measurements suggest 30.2 (SD ± 5.1) predicted metformin binding sites on enteropeptidase, consistent with the small size and positive charge of the drug. The affinity at specific binding site(s) that mediate enzyme inhibition may differ from the K_D_ measured across binding sites by SPR. Nevertheless, enzyme inhibition is likely transient and confined to segments of chyme in transit with the drug.

The metformin dose used in the *in vivo* studies here and in previous reports (4, 7) produces slightly higher peak duodenal metformin concentrations (∼90 mM) compared to the estimates of the human PBPK model. Individuals with lower weight and/or higher metformin doses likely experience increased duodenal metformin concentrations, and our *in vitro* and *ex vivo* studies indicate that 25 mM metformin is sufficient to significantly inhibit enteropeptidase and trypsin activity.

Protein maldigestion could contribute to multiple aspects of metformin therapy. First, metformin use is associated with mild weight loss and mild to severe gastrointestinal side effects. Digestive enzyme inhibition has been employed clinically to treat obesity (*i.e.*, orlistat) and type 2 diabetes (*i.e.*, acarbose), and gastrointestinal side effects can occur with these agents. In animal models, camostat can reduce body weight and improve metabolic disease (8). A more potent and selective enteropeptidase inhibitor, SCO-792, also causes weight loss in animal models (14, 15). The effect of this enteropeptidase inhibitor on weight is attributed to reduced food intake, which is also suggested for metformin (14, 15, 16). There are multiple potential mechanisms for metformin-induced weight loss. We speculate that metformin and other enteropeptidase inhibitors could share a common weight loss mechanism, through which increased nutrient flux into the distal intestine might activate appetite-reducing feedback mechanisms. This could contribute to the apparent paradox that maldigestion reduces, rather than increases, food intake in mice. Second, metformin-mediated microbiome disruption in humans is only partially recapitulated *in vitro* (17), implicating host processes. SCO-792 also disrupts the gut microbiome (14). Third, long-term metformin treatment reduces circulating vitamin B_12_ levels (18). Because trypsin activity is critical for vitamin B_12_ absorption (11), reduced trypsin activity leading to impaired haptocorrin degradation may represent a mechanism for metformin-induced vitamin B_12_ deficiency. Thus, metformin-induced maldigestion may contribute to the complex and dose-limiting intestinal effects of this widely used medication.

## Experimental procedures

### Pharmacokinetic modeling

ADMET Predictor (version 10.4) was used to calculate metformin properties based on the 2-dimensional structure. Results were imported into GastroPlus (version 9.8.2), and a compartmental model was developed based on these predicted drug properties (solubility 100.05 mg/ml, mean precipitation time of 900s, diffusion coefficient 0.75, drug particle density 1.2 g/ml, Log P −0.82). Additional information from the metformin product insert was incorporated into the GastroPlus model: immediate release formulation, molecular weight 165.63, pKa 12.4. Duodenal permeability was set at 0.27 × 10^4^ cm/s (19). The dose was set at 1000 mg twice daily at 12 h intervals (Fig. 1*A*) or 1000 mg once daily (Fig. 1, *B* and *C*). The dose volume was selected as 250 ml with a body mass of 70 kg (Fig. 1, *A* and *B*) with fasted state physiology (duodenal volume 41.56 ml; jejunum two volume 122.3 ml; ileum three volume 49.83 ml). Duodenal volume in the model varied by body mass (30.48 ml for a 50 kg individual and 48.25 ml for a 90 kg individual, Fig. 1*C*). To evaluate model performance, data were extracted from a study reporting the plasma pharmacokinetics of metformin that included a dose of 1000 mg, twice daily, in 16 healthy adult volunteers with an average body weight of 71 kg (5).

### Enzyme activity assays

All enzyme assays were performed at room temperature. Metformin (Millipore #317240) or saline was incubated with enzyme for 5 min before the addition of substrate. At least three independent experiments were completed for each enzyme assay.

Enteropeptidase activity was measured using recombinant human enterokinase (Sigma #SRP6215) at a final concentration of 10 ng/ml or murine tissue extract (containing endogenous enteropeptidase) diluted to the assay dynamic range. Trifluoroacetate salt, a fluorogenic enteropeptidase substrate (Cayman Chemical) was used at a final concentration of 0.4 mM in buffer containing 50 mM Tris and 2 mM calcium chloride. The substrate was added immediately before the kinetic read at excitation/emission of 380/500 nm.

Trypsin activity was measured using trypsin from porcine pancreas (Sigma #T7409) at a final concentration of 0.2 mg/ml or from murine small intestine lumen samples diluted to the assay dynamic range. Na-benzoyl-L-arginine-4-nitroanaline hydrochloride substrate (Sigma #B3133) was used at a final concentration of 0.1 mg/ml. A standard curve was generated using a serial dilution of p-nitroanaline. Reactions included Tris at a final concentration of 50 mM and calcium chloride at a final concentration of 2 mM. The substrate was added immediately before the kinetic read at absorption wavelength 405 nm.

Chymotrypsin activity was measured using a commercial assay (Sigma #MAK345) according to the manufacturer’s instructions. Briefly, the chymotrypsin positive control was used at a concentration of 1 μl per 50 μl reaction. The included chymotrypsin activator and buffer were used per instructions, and substrate was added immediately before kinetic read at the excitation/emission of 380/460 nm.

Elastase activity was measured using a commercial assay (Invitrogen #E12056) according to the manufacturer’s instructions. Briefly, 200 μl reactions were created using the elastase positive control enzyme (0.012U) and the included 1× buffer. The DQ elastin substrate was added at a final concentration of 25 μg/ml immediately before kinetic read at excitation/emission of 485/530 nm.

### Surface plasmon resonance

Surface plasmon resonance was performed using Biacore T200 Biosensor (GE Healthcare). Recombinant human enteropeptidase or amylase was immobilized to the CM5 chip using amine coupling. Metformin was injected from 2× dilution series (500, 250, 125, 62,5, 31.3, 15.6 mM) or 3× dilution series (330, 110, 37, 12.3 mM). For each dilution series, the measurements were made in triplicate, injecting metformin sequentially or in random order and recording responses on two different cells; thus, the affinity was measured from 24 replicates across two independent experiments. The signal over 500 mM reflected aggregated material and did not generate stable responses. Two blank injections were performed before the analyte injection and a single buffer injection; no regeneration was used. The blank injections were averaged to account for system instability. The data were doubly referenced, *i.e*., the signal generated on cell 1 (reference cell) and the data collected for blank (buffer) injection were subtracted from the results collected on active cells (cells that contained immobilized proteins). Results were fitted into a 1:1 binding model using Biacore T200 Evaluation software (ver. 3.1).

### Animal studies

Experiments using mice were performed using protocols approved by the Yale University Institutional Animal Care and Use Committee. Male and female C57BL/6J mice (Jackson Laboratories) were housed in a specific pathogen-free facility and provided standard mouse chow (Teklad Diets #2018S) *ad libitum*.

Experiments were conducted using 12- to 20-week-old mice; within each experiment, animals were of the same age across treatment groups. Treatment group assignment was randomly applied within cages to avoid individual cage effects. In all experiments, mice were fasted for 2 h before sacrifice to minimize variability in pretreatment gastric contents. For quantification of enteropeptidase activity, untreated tissue samples were obtained by collecting 1 cm portions of tissue from the stomach, duodenum (proximal 1 cm small intestine), jejunum (middle 1 cm small intestine), ileum (distal 1 cm small intestine), and colon (middle 1 cm). These samples were placed in 1 ml PBS, chilled on ice, and homogenized by bead-beating. Supernatants were diluted in PBS to achieve enzyme activity within the dynamic range of the enteropeptidase assay, as described above. Supernatant protein was measured using the Qubit protein assay (Fisher #Q33211) for normalization.

Treatments were administered by oral gavage and included 1 mg/ml ultramarine blue pigment (Kremer #45000) to visually identify treated intestinal contents. This pigment was easily removed after sample collection by brief centrifugation. Treatments contained soy protein isolate (Fisher #ICN90545605) at 600 mg/kg. Metformin (Millipore #317240) was administered at 300 mg/kg. Camostat (Cayman #16018) was used at a dose of 5 mg/kg.

### Metformin measurement by LC/MS

Samples of murine small intestine lumen contents (∼50 mg/mouse) were snap-frozen in liquid nitrogen and then weighed. Samples were resuspended in water (10 μl/mg sample) before bead beating followed by centrifugation at 4 °C at 4000*g* for 20 min. Samples and standards were then diluted 500-fold in water. Samples were extracted by placing 20 μl of sample or metformin standard in 1 ml of acetonitrile: methanol (1:1) along with internal standard (sulfamethoxazole, caffeine, ipriflavone, and yohimbine each at a final concentration of 80 nM), vortexed, then placed at −20 °C for 1 h before vacuum drying. Dry samples were then resuspended in 20 μl water. LC-MS was performed using reversed-phase chromatography (C18 Kinetex Evo column, 100 mm x 2.1 mm, 1.7 mm particle size, Phenomenex) using an Agilent 1200 Infinity UHPLC system and mobile phase A: H_2_O, 0.1% formic acid and B: methanol, 0.1% formic acid. Column compartment was kept at 45 °C. Five microliter of sample were injected at 100% A and 0.4 ml/min flow followed by a linear gradient to 95% B over 5.5 min and 0.4 ml/min. The qTOF (Agilent 6550) was operated in positive scanning mode (50–1000 m/z) with the following settings: VCap: 3500 V, nozzle voltage: 2000 V, gas temp: 225 C; drying gas 13 L/min; nebulizer: 20 psig; sheath gas temp 225 °C; and sheath gas flow 12 L/min. Mass calibration was performed using a reference solution with 121.0509 m/z and 922.0098 m/z. Peak integration was done with MassHunter Quantitative Analysis Software (Agilent, v. 7.0). Metformin was identified (retention time ∼0.63 min, m/z 130.1) and quantified using a standard generated from serial dilution of metformin (concentration ranging from 0 to 100 mM). Extraction was normalized to the average signal of caffeine internal standard.

### Protein hydrolysis

To determine relative intraluminal protein hydrolysis, mice were treated with 60 mg/ml soy protein isolate in combination with saline, metformin, or camostat (total volume 15 μl/g mouse weight) and samples collected from the small intestine 20 min after gavage. Small intestine contents were diluted using 10 μl water/mg contents on ice and Halt protease inhibitor cocktail (Thermo Fisher #78430) was immediately added. After vortexing and centrifugation, the supernatant was passed through a 10 kD filter to select for small proteins and peptides. Each sample (10 μl) was then added to 100 μl OPA reagent. OPA reagent was prepared as described in previous publications (20). Samples were incubated in OPA reagent for 2 min before reading absorbance at 340 nm on a plate reader. The measured absorbance was divided by the total protein content of the sample as measured by the Qubit protein assay (Fisher #Q33211).

### Western blot

For measurement of murine transcobalamin, small intestine contents were frozen in liquid nitrogen at the time of harvest and later thawed and diluted in 10 μl PBS/mg contents. Halt protease inhibitor cocktail (Thermo Fisher #78430) was added to 1× final concentration, and samples were vortexed and centrifuged. The protein content of the supernatant was measured using the Qubit protein assay (Fisher #Q33211) according to manufacturer instructions. As commonly used housekeeping proteins may not be present in constant quantity within the intestinal lumen and may themselves be subject to protease activity, we normalized the loading of Western blots based on total protein content as measured by the Qubit assay. The Qubit assay was found to be unaffected by metformin concentrations as high as 500 mM. Samples were boiled in loading dye containing 2.5% 2-mercaptoethanol, and 150 μg total protein was loaded per well in a gradient (4–12%) bis-tris gel (Thermo Fisher #NP0323BOX). After transfer, blots were probed with 1:2000 rabbit anti-murine transcobalamin antibody (Novus Biologicals #NBP17422020UL).

For measurement of exogenous recombinant human haptocorrin degradation by murine small intestine samples, small intestine contents were extracted as described above but without the addition of protease inhibitors. Recombinant human haptocorrin/transcobalamin 1 (Biomatik #RPC24978)) was diluted to 330 ng/μl in a buffer containing 50 mM Tris and 2 mM CaCl_2_. This was incubated at a final haptocorrin protein concentration of 220 ng/μl with an aqueous extract of murine small intestine samples (250 μl/mg sample) at 25 °C for 5 min before quenching with Halt protease inhibitor. Quenched incubations were then boiled in a loading buffer containing 2.5% 2-mercaptoethanol, and a Western blot was performed as described above and probed with 1:1000 rabbit anti-human transcobalamin one antibody (Novus Biologicals #H00006947-D01P). Image quantification was done using ImageJ 1.53 t to calculate the background-subtracted signal intensity from each band.

### Statistics

Statistics were performed with GraphPad Prism (version 9.4.1) using one-way ANOVA with Dunnett’s multiple comparison test.

## Data availability

All study data are included in the article.

## Conflict of interest

The authors declare no conflicts of interest with the contents of this article.
